# Implantable cardioverter-defibrillator shocks and nonsustained rapid ventricular rhythms

**DOI:** 10.1007/s12471-022-01747-y

**Published:** 2022-12-20

**Authors:** H. Witteveen, P. Stellingwerf, H. F. Groenveld

**Affiliations:** Department of Cardiology, University Medical Centre Groningen, University of Groningen, Groningen, The Netherlands

## Answer

**Fig. 1:**
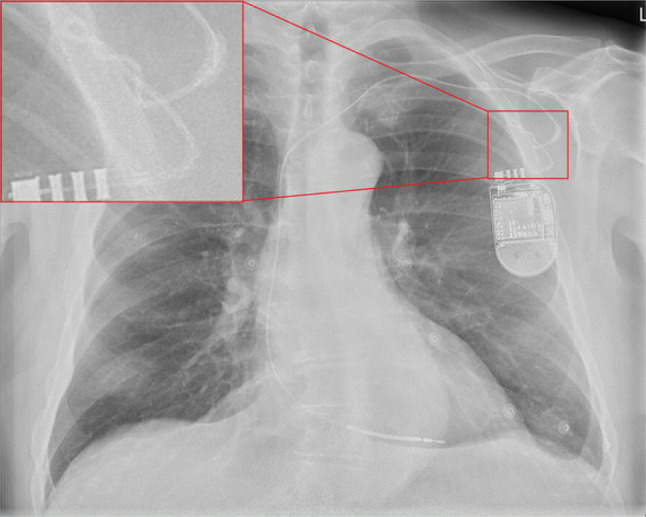
Same chest radiograph as shown in Fig. 1 in the question, with enlargement of proximal part of ventricular lead of the implantable cardioverter-defibrillator

The ECG (Fig. 1 in the question) shows sinus bradycardia and 8 fast ventricular pacing spikes with ventricular capture. The chest X‑ray depicts a normal position of the ventricular lead but a coiled proximal part of the lead (Fig. 1). Coiling makes the lead prone to lead fracture. Compared with the chest radiograph that was taken immediately after implantation of the ICD, not only was the lead coiled, but the pacemaker generator was also twisted.

**Fig. 2:**
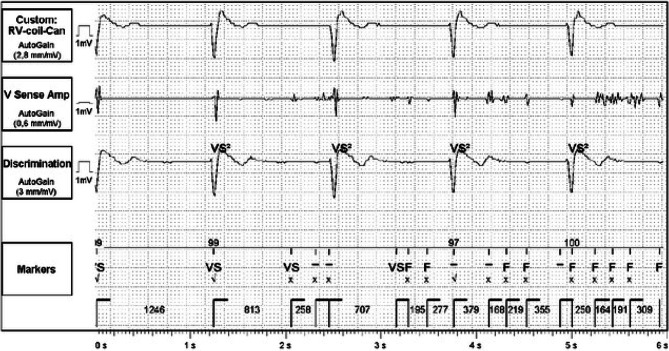
Implantable cardioverter-defibrillator tracing: the V sense shows noise, which was annotated as ventricular fibrillation

ICD interrogation revealed several ventricular tachycardia/ventricular fibrillation episodes and 3 inappropriate shocks due to noise. Fig. 2 illustrates an ICD tracing of the noise, which was annotated as ventricular fibrillation. The device responded with anti-tachy-pacing, which can be seen on the ECG (Fig. 1 in the question), and eventually inappropriate ICD shocks.

This patient experienced inappropriate ICD shocks, which were caused by a twisted ICD that led to a lead defect. This phenomenon is called Twiddler’s syndrome and was first described by Bayliss et al. in 1968 [1]. The syndrome is rare—estimated to occur in 0.07%–7% of implanted devices—and usually strikes in the first year following pacemaker implantation [2, 3]. The trigger for lead coiling is unintentional or deliberate manipulation of the device generator within its pocket; our patient denied manipulation of the device.

The problem was solved by implanting a new ventricular lead. Additionally, the size of the pacemaker pocket was reduced. To prevent the ICD device from turning, suture fixation of the pulse generator was performed.
